# Temporal context-guided memory capabilities in rodents

**DOI:** 10.1038/s41598-025-95410-2

**Published:** 2025-05-28

**Authors:** Benjamin J. A. Slater, Christopher I. Petkov, Alexander Easton

**Affiliations:** 1 Biosciences Institute, Newcastle University, Newcastle Upon Tyne, NE2 4HH UK; 2 Department of Neurosurgery, University of Iowa, Iowa City, IA 52242 USA; 3 Department of Psychology, Durham University, Durham, DH1 3LE UK

**Keywords:** Cognitive control, Decision, Intelligence

## Abstract

Environmental contexts serve as powerful cues for episodic memory, allowing humans to recall events tied to specific settings. While rats can learn context-specific associations and temporal order, their ability to manage multiple contexts and rapidly adapt to changes in context remains unclear. This study investigated whether rats could order objects across two distinct contexts. Eight Lister Hooded rats were trained in a dual-context maze, where each context contained a pair of objects. In each trial, rats entered the maze, selected an object, and then re-entered either the same or a different context to complete the trial in the correct temporal order. Six rats successfully learned object order within a single context, but only two reached criterion in the more complex two-context condition. Group error analyses revealed a partial reliance on a procedural learning strategy and a tendency to favour one context, where prior location influenced object selection in subsequent trials. While two rats successfully adapted to the two-context condition beyond these simple strategies, most struggled with context switching, exhibiting perseveration difficulties—a trait also observed in some humans. These findings highlight the evolutionary foundations of context-guided memory and reveal remarkable individual variability in the ability to flexibly navigate multiple contexts.

## Introduction

Our ability to remember day-to-day experiences often relies on associating events with the contexts in which they occur^1,2^. For example, recalling your commute to work—whether by bicycle or by car—might also bring to mind preceding events, such as eating breakfast, and subsequent ones, like greeting colleagues or heading to the coffee machine. Although these events unfold across distinct settings (home, travel, work), they are linked by transitions and the passage of time. Context not only organises our memories into cohesive episodes^3^ but also guides our behaviour^4^; for instance, arriving early at work might influence whether you seek to greet colleagues or start working immediately. Such context-dependent processes are critical for adapting behaviour in changing circumstances, particularly when contexts shift^5^. By flexibly responding to shifting environments, humans can navigate complex surroundings, anticipate outcomes, and adjust their behaviour in response to changes in context, sensory sequences, and the passage of time^6–8^.

Human episodic memory is particularly adept at recalling events across multiple locations^9,10^, an ability likely shaped by evolutionary pressures on primates for navigation and foraging. Some of these cognitive capacities may also be shared with more distantly evolutionarily related extant animals^11–14^. For example, research has shown that rats can disambiguate overlapping sequences by using contextual cues^15,16^, suggesting a broadly conserved ability to form schemas—neural frameworks that integrate new memories and update existing ones^17–19^. Although rodents are known to form context-dependent associations (such as linking a specific environment with an aversive event^20–24^), it remains unclear whether they can flexibly adapt their behaviour when contexts change. This raises an important question of whether rodent schemas support the integration of multiple contexts and the rapid adaptation of behaviour to contextual shifts, as seen in human episodic memory.

To address this epistemic gap, we built on the work of Eichenbaum and colleagues^25,26^, who demonstrated that rats could use contextual cues to disambiguate overlapping objects. We developed a two-context task designed to assess whether rodents could learn and recall associations between object-pairs and their respective contexts. In this task, rats were required to adapt to mid-trial context changes; rather than simply initiating responses in a new context, the rats’ behavioural choice needed to accommodate the passage of time.

In this novel task, rats were presented with two distinct objects in each of two contexts defined by the colour and pattern of the testing box. They were initially trained to choose objects in a specific order based on the context they were in. During each phase, their ability to reach and maintain a predefined criterion performance was evaluated before being tested in progressively more complex aspects of the task—how they adapted their responses to the object-pairs when the context changed mid-trial. Drawing on human studies^27,28^ that examined how individuals adapt behavioural selection of multiple objects across contexts, we hypothesised that object-pairs confined to a single context would be learned and recalled more easily than those spanning multiple contexts, as the latter demands greater cognitive flexibility.

## Results

We first present the primary results from individual animal criterion performance (Primary results). This is followed by the secondary results from group analyses at each testing phase for all six of the rats that progressed from the initial testing phase (Secondary analyses). The group analysis of rodent error rates offers insights into the group of rodents’ likely strategies during the task, limited by the sample size in the study cohort.

### Primary results: individual criterion performance

**Fig. 1:**
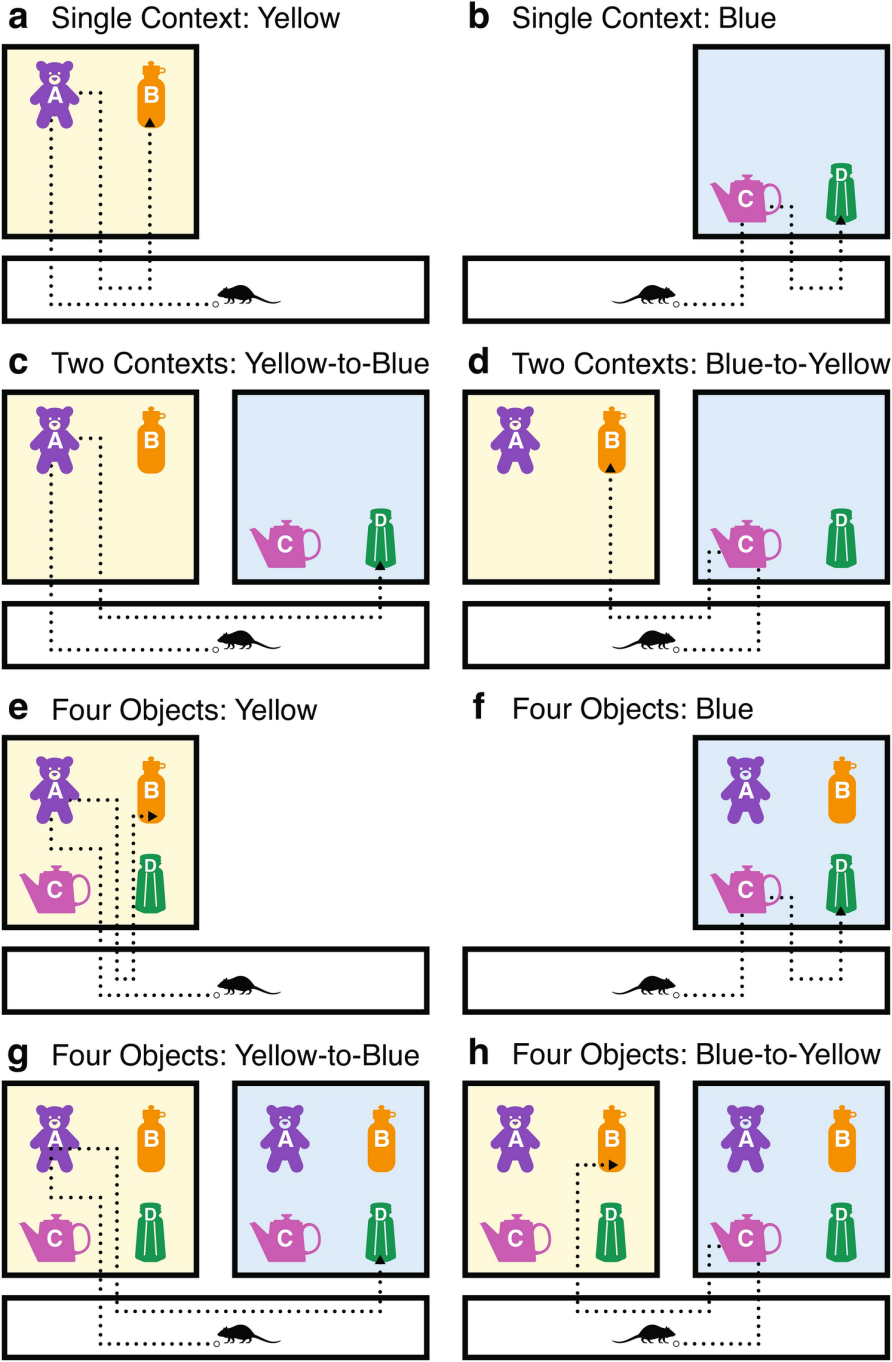
Schematic representation of testing stages and object-pairs across single and two contexts. (**a**) and (**b**) The correct object order in the Single Context—Two Object-pairs phase (Phase 1), with (**a**) the correct ordering in the Yellow context and (**b**) in the Blue context. (**c**) and (**d**) The correct object order in the Two Contexts—Two Object-pairs phase (Phase 2), with (**c**) representing the transition from Yellow-to-Blue context, and (**d**) the transition from Blue-to-Yellow context. (**e**)–(**h**) The four possible configurations for testing, with all objects (A, B, C, D) present in either context during Phase 3 (Two Contexts—Four Object-pairs). These configurations reflect the combinations of Single Context trials (**e**) and (**f**) or Two Context trials (**g**) and (**h**).

The experiment was divided into three phases, each progressively increasing the cognitive demands on the rats to adapt their responses to pairs of objects within single, then multiple, contexts. The three phases are illustrated in Fig. 1. Phase 1, the Single Context—Two Object-pairs phase, assessed whether rats could learn to recall the correct temporal order of pairs of objects within a single context (Fig. 1a,b). Phase 2, the Two Context—Two Object-pairs phase, required the rats to remember pairs of objects across two different contexts (Fig. 1c,d). Phase 3, the Two Context—Four Object-pairs phase, tested rats that progressed to Phase 2 on whether they could correctly adapt to a change in context mid-trial with all object choices (both pairs) available (Fig. 1e–h).

#### Phase 1: Single context object-pair learning

Phase 1 assessed whether the eight starting rats in the study could recall the correct order of two objects when presented within a single context, defined by the background colour—yellow or blue (Fig. 1a,b). The maze consisted of two distinct contexts connected by a corridor. Each context contained four food wells, but only two were covered by objects in each trial. To simplify learning, object locations were fixed in each context. In the yellow context, Object A was positioned in the top-left corner and Object B in the top-right corner. In the blue context, Object C was in the bottom-left corner and Object D in the bottom-right corner. Each trial began with a rat entering one context and selecting an object (e.g., Object A in the yellow context). The rat then shuttled out and re-entered to choose the second object in the pair (e.g., Object B). Object selection involved pushing the object aside to reveal a food pellet underneath. To prevent odour-based cues from influencing choices, both wells were baited, ensuring that selection was based on learned associations rather than scent. Incorrect choices led to immediate removal to a “time-out” cage for two minutes. Each rat completed six trials per day, with trials counterbalanced across contexts to minimise bias.

**Fig. 2:**
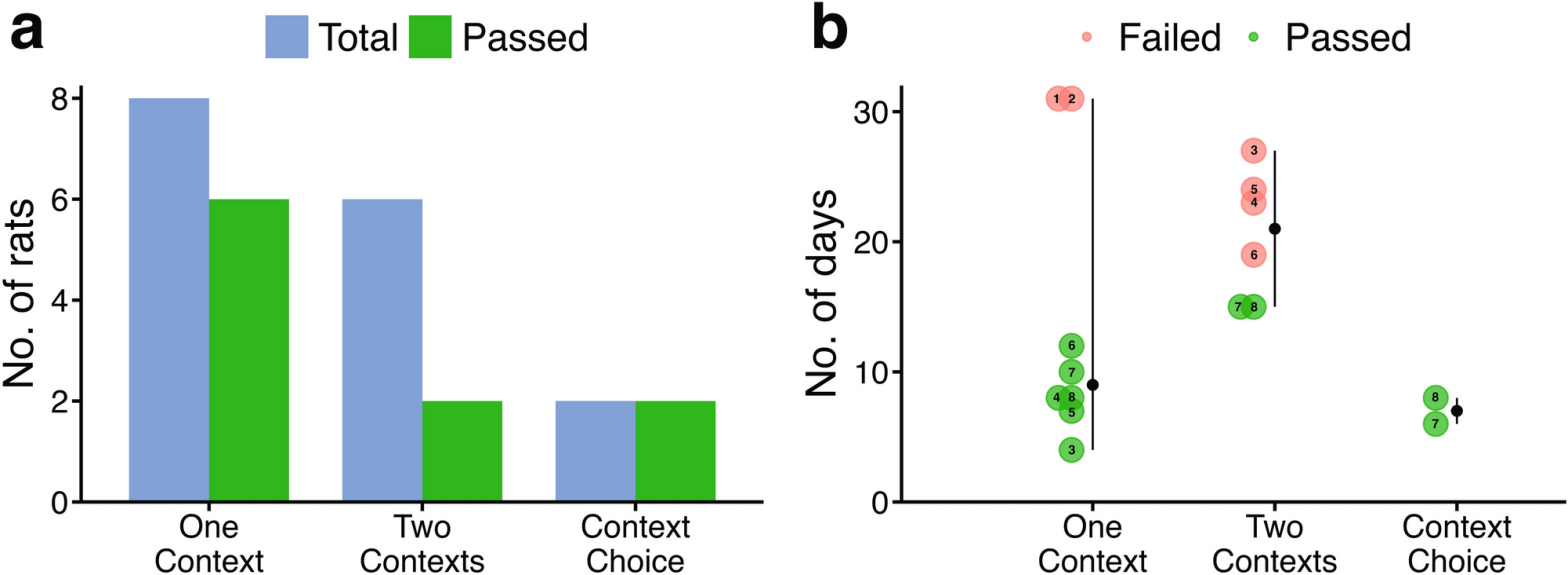
Number of rats that passed each testing phase and the time taken to reach criterion.  (**a**) The total number of rats tested at each phase (blue bars) and the number that successfully met the predefined criterion (green bars). The criterion for success was achieving 10 correct responses out of 12 trials (83% correct) over two consecutive days (*p* = 0.019, binomial test, one-tailed). (**b**) The median number of days spent on each testing phase, with rats that successfully completed the phase (green) and those that did not reach criterion (red) identified. Testing was capped at a maximum of 31 days for all rats, regardless of their progression. ID of each rat is provided with numbers 1 to 8.

To reach criterion performance, a rat had to achieve 10 correct responses out of 12 trials over two consecutive days. This threshold (83% correct) was set to ensure that performance significantly exceeded chance levels (6 out of 12 trials, or 50%) and was confirmed with a binomial test (*p* = 0.019, one-tailed). Progression to the next phase was allowed only if a rat met this predefined criterion. Testing was capped at 31 days to ensure consistency across subjects. Of the eight rats tested, six (6/8 = 75%) met criterion in Phase 1, but only two (2/8 = 25%) of those six succeeded in Phase 2 (Fig. 2a). Both rats that progressed to Phase 2 also reached criterion in Phase 3.

The average number of days undertaken by each rat to reach criterion for Phase 1 (Single Context phase) was 14 days (mean: 13.9 ± SD: 10.8), with the two rats that failed taking the full experimental time (31 days) in attempting to reach threshold performance (Fig. 2b).

#### Phase 2: Adapting to change in context—two objects only

The six rats out of eight that reached criterion performance in Phase 1 progressed to evaluation in Phase 2, where they had to adapt to a change in context. Only two objects were available at a time as shown in Fig. 1c,d. Each trial began in one context, where the rat had to choose the correct object before transitioning to the other context to complete the object-pair. Figures 1c,d display the two possible object-pairs the animals could encounter. For example, if transitioning from the yellow context to the blue context, rats had to choose Object A first in the yellow context, followed by Object D in the blue context (Fig. 1c). The reverse transitionfrom the blue context to the yellow context—required choosing Object C first in the blue context,  followed by Object B in the yellow context (Fig. 1d).

Learning these between-context object-pairs proved significantly more challenging for the remaining cohort of rats. Only two out of six rats tested successfully reached criterion (Fig. 2a). On average, it took 20 days to reach criterion (mean: 20.5 ± SD: 4.97), with the two successful rats completing the phase in just 15 days (Fig. 2b).

#### Phase 3: Adapting to change in context—all four objects

In the final phase, the two successful rats from Phase 2 were tested on their ability to freely choose a starting context while maintaining the correct object order. To increase complexity, both contexts now contained all four objects (A, B, C, and D) instead of just two, introducing the distractor object pair (Fig. 1e–h). The rats could choose which context to enter first, and the required object pairing was determined by this choice.

Both rats successfully reached criterion within 7 days (mean: 7.00 ± SD: 1.41; Fig. 2a,b), completing this phase significantly faster than earlier stages. This demonstrates their ability to effectively utilise all learned object-pairs, adapt flexibly to changing contexts, and overcome the additional challenge posed by distractor objects. Individual performance (Supplementary Fig. 1) revealed that learning was not strictly linear—performance occasionally declined before reaching criterion, suggesting a dynamic learning process rather than a steady progression.

### Secondary analyses

Secondary analyses for the cohort of rats were conducted to explore factors contributing to the rats’ difficulty in learning object-pairs across contexts. These cohort error analyses aimed to identify the presence of behavioural strategies, such as procedural learning (a left–right object location strategy), context sensitivity, and how prior trials, single or multiple contexts, and objects affected error rates.

**Fig. 3:**
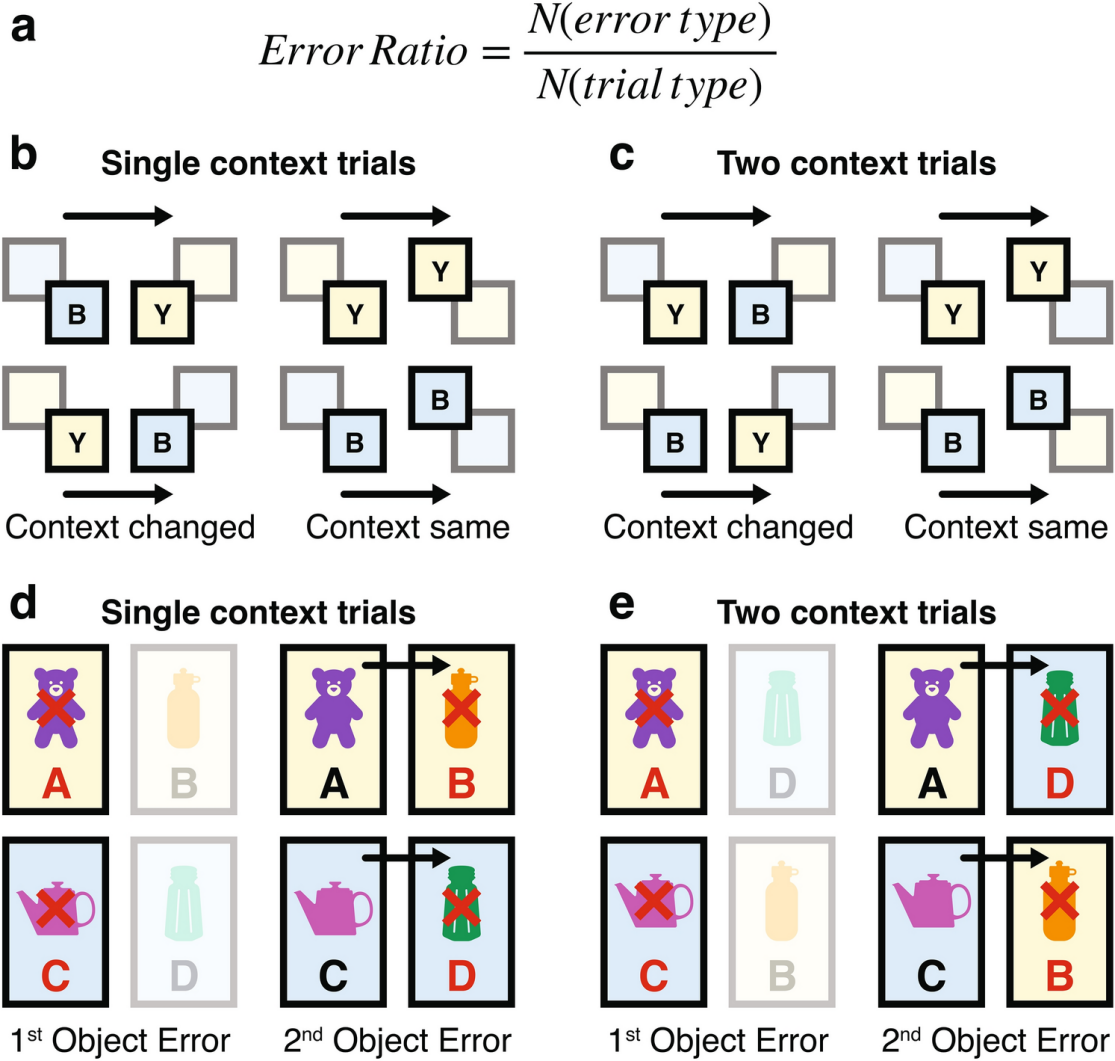
Examples of error ratio for each error and trial type. (**a**) Error ratios were calculated based on the number of errors divided by the total number of trials attempted. (**b**) and (**c**) The categorisation of error types in Single Context and Two Context trials, respectively, focusing on context transitions between trials. Specifically, context change was defined by comparing the context in which the preceding trial ended to the context where the subsequent trial began. For example, if the first trial ended in the Yellow context and the next trial began in the Blue context, this transition was classified as a ‘change’ in context, even if the overall trial order itself remained the same (i.e., going from a Blue-to-Yellow trial to a Blue-to-Yellow trial). (**d**) and (**e**) Error types in Single Context and Two Context trials, respectively, focusing on object selection. Here, errors were further differentiated into ‘1st Object’ and ‘2nd Object’ errors, based on which object within the object-pair the rat chose incorrectly.

Error ratios were calculated for each rat by dividing the number of errors by the total number of trials attempted in each testing phase (Fig. 3a). This allowed for direct comparisons of group performance at each phase.

To examine the impact of context change, trials were categorised based on whether the context of the preceding trial had changed or remained the same (Fig. 3b). A trial was classified as “context changed” if it occurred in a different context from the previous trial (e.g., switching from Blue to Yellow) and “context same” if both trials occurred in the same context (e.g., both trials in Blue). By structuring trials in this way, we assessed whether overall performance was influenced by residual contextual information from previous trials.

### Does context change between trials influence performance at the group level?

To explore the factors contributing to increased errors in the cohort of rats, we analysed trials using a linear mixed-effects model. Trials were ordered sequentially for each rat across all sessions, comparing the context of the current trial with that of the previous trial (Fig. 3b). In the Two Context phase, where two contexts were presented per trial, we compared performance on the last context of a trial with the starting context of the next trial (illustrated in Fig. 3c). This approach allowed us to determine whether rats adapted to the context changes (indicating low error rates) or whether certain factors—such as biases toward prior actions or previous object locations – contributed to increased errors.

All rats that participated in Phase 1 (Single Context phase, *n* = 8) and Phase 2 (Two Context phase, *n* = 6) were included in the analyses. Additionally, errors were categorised as ‘1st Object’ and ‘2nd Object’ errors, corresponding to the object within the object-pair on which the rat failed (Fig. 3d,e). This classification was applied to both Single Context and Two Context trials to further examine how stable versus changing contexts within and between trials influenced performance.

### Single context trials

For the Single Context phase (Phase 1), we applied a linear mixed-effects model with error ratio as the dependent variable. Error type (1st or 2nd Object errors) and context change (changed or same) were included as fixed effects, with a random intercept for individual rats to account for variability. An interaction term (error type × context change) was also included to assess whether context change influenced error rates differently depending on error type.

**Fig. 4:**
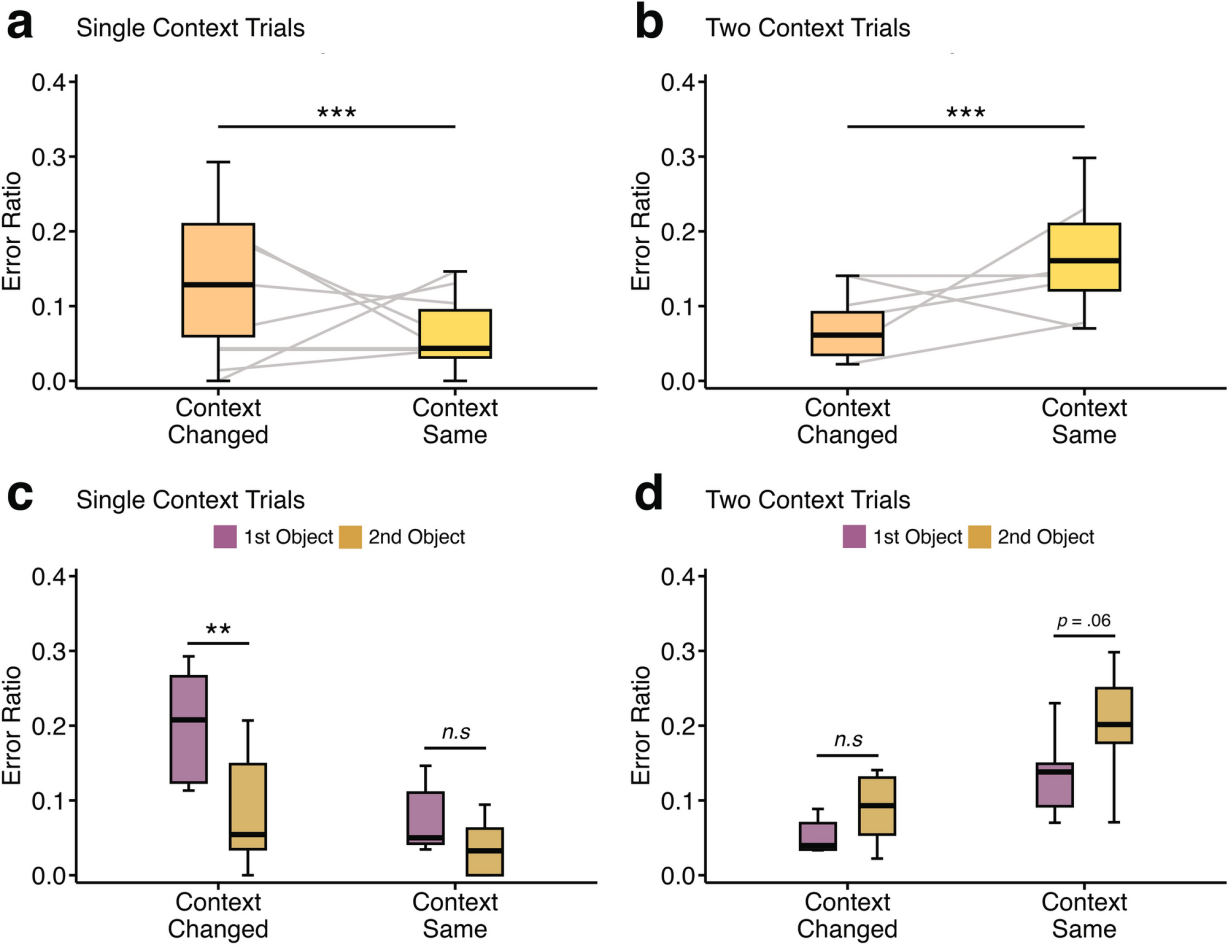
Error ratios for Context Changed vs. Context Same trials by individual rat. Error ratios based on whether the preceding trial context changed (‘Context Changed’) or remained the same (‘Context Same’) when trials were ordered by individual rat. (**a**) Error ratios for Single Context trials, showing significantly higher errors when the context changed compared to when it remained the same (*p* < .001). (**b**) Error ratios for Two Contexts trials, showing significantly lower errors when the context changed compared to when it remained the same (*p* < .001). Individual animal trajectories are represented by lines connecting paired data points, illustrating within-subject performance. (**c**) Error ratios for ‘1st Object’ and ‘2nd Object’ errors for Single Context trials. Rats made significantly more 1st Object errors when the context changed (*p* < .01), but there was no significant difference when the context remained the same. (**d**) Error ratios for ‘1st Object’ and ‘2nd Object’ errors for Two Context trials. No significant differences were observed, although a statistical trend showed increased ‘2nd Object’ errors when the context remained the same (*p* = .06).

The analysis revealed a significant main effect of context change, with higher error rates when the context changed (mean: 0.14 ± SD: 0.10) compared to when it remained the same (mean: 0.06 ± SD: 0.05; *F* (1, 21) = 15.7, *p* < 0.001, Fig. 4a). This suggests that rats struggled to update contextual information between trials and instead tended to persist with previous trial actions when faced with a contextual change.

Post-hoc analysis revealed that when the context remained the same, errors for the first and second object were low and did not significantly differ (*t* (21) = 1.17, *p* = 0.255, *d* = 0.585, type-III hypothesis test, Fig. 4c). However, when the context changed from that present on the prior trial, rats were significantly more likely to make an error on the first object (mean: 0.20 ± SD: 0.07) than on the second object (mean: 0.09 ± SD: 0.08; *t* (21) = 3.62, *p* < 0.001, *d* = 1.81, type-III hypothesis test, Fig. 4c).

This pattern is consistent with the rats repeating their previous trial action rather than adapting to the change in context. Specifically, when the context changed, rats made significantly more errors choosing the first object, indicating they were heading directly to the second object instead. Given that the second object was always positioned on the right side, this behaviour is consistent with perseveration to the last rewarded location or adherence to a procedural learning rule (i.e., choosing the location of the object in the prior context).

However, this pattern was different when the context remained the same. In these cases, error rates were lower and there was no significant difference in first and second object selection errors, implying that rats were more likely to correctly choose the first object and effectively ‘restart’ the new object-pair order.

### Two context trials

For Two Context trials, a similar linear mixed-effects model was used, but the pattern of results was reversed from that of the Single Context trials considered in the previous section. Here, error rates were significantly *lower* when the context changed (mean: 0.07 ± SD: 0.04) than when it remained the same (mean: 0.17 ± 0.08;* t* (15) = − 4.32, *p* < 0.001, *d* = − 1.73, type-III hypothesis test, Fig. 4b). This finding suggests that, under Two Context conditions, errors were more likely to occur when the context was stable between trials. Unlike in the Single Context phase, where errors increased following a context change, in Two Context trials, errors were more frequent when the context remained the same, suggesting a shift in strategy relative to the Single Context data.

The rats’ behavioural errors on the Two Context trials suggest that context changes influenced their decision-making process. Specifically, when the context remained the same between trials, rats tended to initiate the trial correctly, showing a statistical trend of being more likely to choose the first rather than the second object (*t* (15) = 2.06, *p* = 0.058, *d* = 1.19, type-III hypothesis test, Fig. 4d). These observations suggest that the rats that progressed to the Two Context phases were better able to identify the start of a new trial but struggled to adjust their response for the second object when the context changed within the current trial.

### Is there a bias towards certain contexts?

To examine how specific contexts influenced error types, error rates in individual contexts (Yellow and Blue) across Single Context and Two Context trials were analysed. This analysis aimed to determine whether rats’ performance was affected by the specific context they were in and whether their behavioural strategies differed within and between contexts. Data from all rats were included (*n* = 8) for Single context trials or the six rats (*n* = 6) that progressed to Two Context trials, with errors grouped by individual rats in each context.

For the Single Context phase, a linear mixed-effects model was used, with error ratio as the dependent variable and error type (1^st^ or 2^nd^ Object errors) and context colour (Yellow or Blue) as fixed effects. A random intercept for each rat accounted for individual variability. The model also included an interaction term (error type × context colour) to determine whether the effect of context colour on error rates differed by error type.

**Fig. 5:**
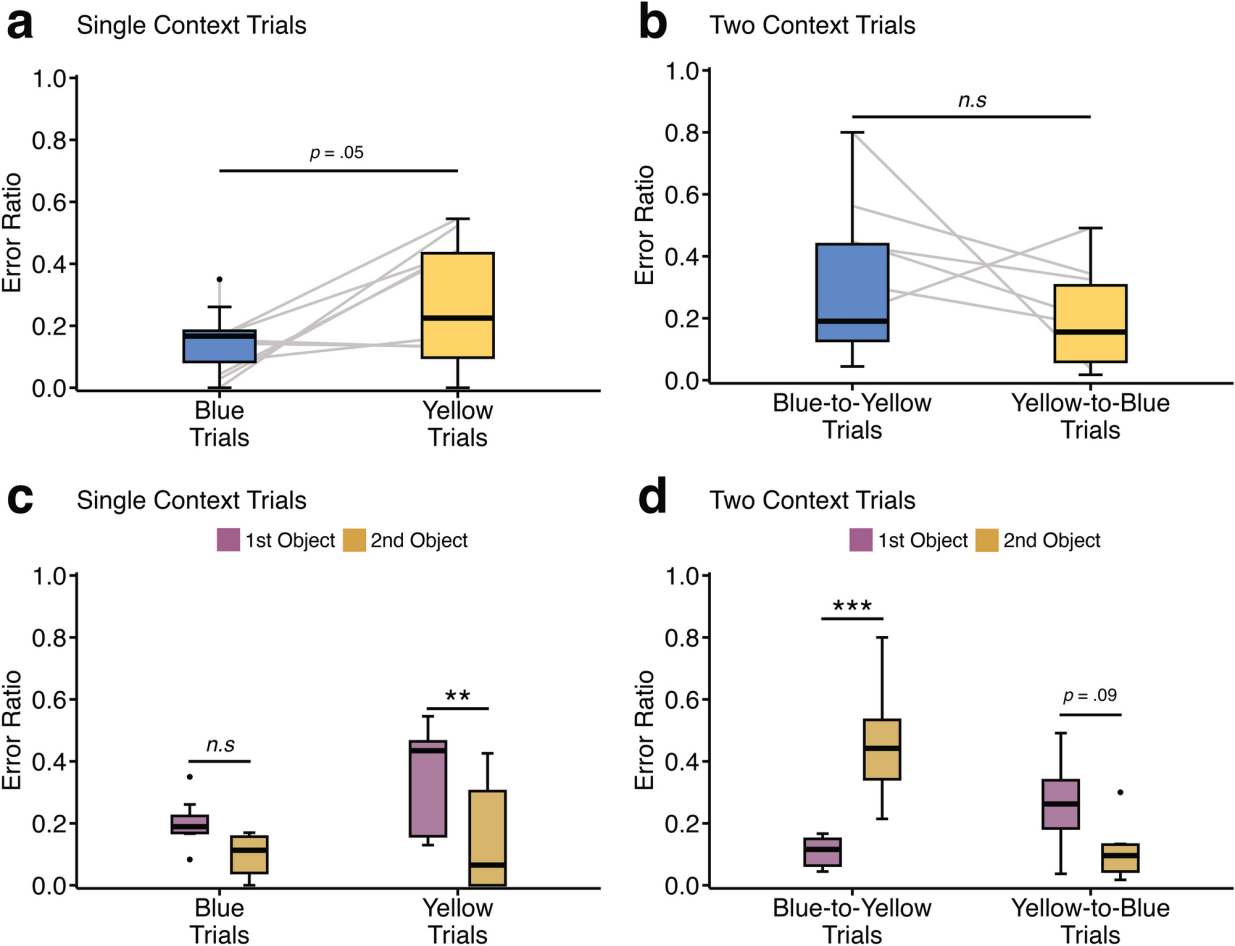
Error ratios between Blue and Yellow trials in Single and Two context trials ordered by individual rat. Error ratios and error type comparisons for Blue and Yellow trials in Single Context and Two Context phases. (**a**) No significant difference in overall error ratios between Blue and Yellow trials during the Single Context phase (*p* = .051). (**b**) In the Two Context phase, no overall difference in error ratios was found between Blue-to-Yellow and Yellow-to-Blue transitions (*p* = .113). Individual animal trajectories are represented by lines connecting paired data points, illustrating within-subject performance. (**c**) During Single Context trials, rats made significantly more ‘1^st^ Object’ errors than ‘2^nd^ Object’ errors for Yellow trials (*p* = .008), but no significant difference was observed for Blue trials (*p* = .144). (**d**) A significant increase in ‘2^nd^ Object’ errors were observed for Blue-to-Yellow trials compared to '1^st^ Object’ errors (*p* < .001), while no significant difference was found for Yellow-to-Blue transitions (*p* = .09).

In Single Context trials, there was a statistical trend toward higher error rates in the Yellow context compared to the Blue context (*t* (21) =− 2.07, *p* = 0.051, *d* =− 0.733, type-III hypothesis test, Fig. 5a), suggesting that the Yellow context trended towards posing greater difficulty for the rats.

In the Yellow context, rats made significantly more first object errors (mean: 0.35 ± SD: 0.18) compared to second object errors (mean: 0.15 ± SD: 0.18; *t* (21) = 2.92, *p* = 0.008, *d* = 1.46, type-III hypothesis test, Fig. 5c). No significant difference in error type was observed in the Blue context (*p* = 0.144, Fig. 5b), although the mean differences appear to follow a similar trend (Fig. 5c).

In Two Context trials, error rates did not differ under the two contexts (*t* (15) = 1.29, *p* = 0.113, *d* = 1.69, type-III hypothesis test, Fig. 5b). However, when analysing error types, rats showed greater difficulty in the Yellow context, particularly when it was the final context of a trial. Second object errors significantly increased in Blue-to-Yellow trials (*t* (15) = 4.33, *p* < 0.001, *d* = 2.50, type-III hypothesis test, Fig. 5d), whereas the opposite pattern was a statistical trend in the reverse transition (Yellow-to-Blue; *p* = 0.088, Fig. 5d). This suggests that while rats could initiate object-pair choices in the Yellow context, they struggled to complete them in the Yellow context.  This difficulty might explain why only two rats were able to advance through all testing phases. The overall pattern of results is consistent with context sensitivity particularly in the Two Context conditions. Unlike the Single Context phase, which can be explained by a procedural learning rule (e.g., last object location or left–right alternation strategy), the Two Context data are difficult to explain using such strategies (Figs. 4, 5). These observations also suggest that the rats did not appear to be strictly following a procedural rule—such as a left–right alternation strategy, switching sides for reward retrieval upon each entry into any context.

## Discussion

Human episodic memory excels at providing context to disparate memories and removing ambiguity from overlapping events under different contexts^29^. While context-dependent memory is well-documented in humans, its flexibility in nonhuman species, particularly rodents, remains an open question. Building on prior research in context-guided decision-making in rodents, we tested whether rats could adapt to and manage with at least two contexts that could change between and within trials. Our findings revealed substantial individual variability: while six out of eight rats successfully learned object-pair order in a single context, only two reached the predefined criterion when context changed within a trial, and all four objects were available for them to choose from. This provides initial support for context sensitivity in some rats that is more like human abilities, with the caveat that most rats showed perseveration problems or less robust context sensitivity that did not appear to flexibly transfer to all of the contexts.

Previous research has demonstrated that rats are adept at associating aversive stimuli with a contextual environment and are capable of deciphering object-context associations, even when objects are present in more than one context^25,26^. These studies, however, often relied on rats choosing single objects or stimuli, used a limited set of contexts, or did not assess rats’ abilities to adapt to contextual changes within a trial. In the current study, we tested the ability of Lister Hooded rats to remember two object-pairs whose temporal order depended on context, in our case, whether the testing context was blue or yellow in colour. The task implemented a dual context maze, within which were up to four objects whose identity and location were fixed for both contexts. Animals could benefit from the fixed location of the objects but had to rely on the context they were in or adapt to a change in context within the testing trial to select the correct order of objects.

Our primary results are based on individual animal criterion performance. While most rats demonstrated the ability to learn the temporal order of objects within a single context, many displayed a difficulty in adjusting previously obtained knowledge to accommodate the expected order of known objects across multiple known contexts or when the context changed mid-trial. Of the eight rats tested, six were successful in reaching threshold performance when the object-pair remained within a single context. When the same six were subsequently tested on completing a pair that involved a change in context mid-trial, only two were successful in learning this more complicated phase. Those two animals also reached performance criterion on the context change conditions with all four objects available.

Our secondary analyses focused on evaluating the behavioural errors across the entire cohort, allowing us to identify the strategies adopted during the Single and Two Context phases. Specifically, we examined the effect of context change between trials, assessing performance both when the context of the current trial matched that of the prior trial and when it differed.

We observed striking differences in error rates between the Single and Two Context phases, consistent with a shift in strategy under the context change conditions for the six rats that progressed beyond the Single Context phase (see Fig. 4). In the Single Context trials, rats struggled with between-trial updating, often repeating their last action from the previous trial when the context changed. This pattern of errors aligns with behavioural perseveration relative to the prior trial or a procedural strategy, wherein the rodents returned to the last rewarded object location. In contrast, during the Two Context phase—where the context changed within the trial—the same six rats adapted their strategy. They exhibited fewer errors when the context changed compared to when it remained the same, showing the opposite pattern of errors from those observed in the Single Context trials. This reversal of error patterns between trial types suggests a context-sensitive shift in decision-making, although it was only a useful strategy for the two rats that completed all phases.

Moreover, in the Two Context phase, errors for the first and second objects occurred in the opposite direction to those in the Single Context phase (compare Fig. 4c to d), suggesting that the rats adjusted their responses based on the prior trial’s context. However, when the context changed again within a trial, the rats exhibited increasing difficulty selecting the correct second object, often selecting the incorrect second object even after correctly choosing the first object (Fig. 4d). This indicates that, for the rats that progressed, the winning response strategy shifted towards one that was more context-sensitive and able to facilitate progression to the Two Context phase. Nevertheless, only two of the rats successfully progressed to Phase 3, which involved presenting two pairs of objects under either context.

Finally, we observed that while all six rats conducting the Two Context trials could initiate object-pair choices in the Yellow context, they struggled to complete them within the same context. This bias towards the Blue context, along with higher error rates in the Yellow context, may have hindered all but two of the rats that advanced through all of the testing phases. Overall, the pattern of results supports the notion of context sensitivity under the Two Context condition, a pattern that, unlike performance in the Single Context phase, cannot be readily explained strictly by a procedural learning rule, such as selecting the last rewarded object location or employing a left–right switching strategy (compare Figs. 4 and 5).

In considering how rats might overcome this difficulty in flexibly adapting to changing contexts to better recall temporal order across multiple contexts, a possible approach is for the rats to update a memory sequence when the context changes, rather than relying on the context change as a cue to reinstate the memory sequence from the beginning. The latter strategy would only hinder their performance.

Crystal and Smith^30^ raised the possibility that rats can form bound representations of episodes involving multiple features and contexts, which are resistant to interference from multiple objects and can persist over long retention intervals. They suggest that similar mechanisms facilitate the handling of overlapping episodic memories by integrating features such as ‘what’, ‘where’, ‘source’, and ‘context’. We extend Crystal & Smith’s proposal with the results obtained here, which suggest that some rats also appear to accommodate contextual change, particularly when it requires not starting with the first item under that particular context. Namely, in the present study, when the two rats that excelled at all phases entered the Blue context and began the trial, they were able to identify the second object when the context changed (i.e., when the rat moved to the Yellow context), rather than the first object under that context, as they had to in the Single Context phase (see Fig. 1d).

**Fig. 6:**
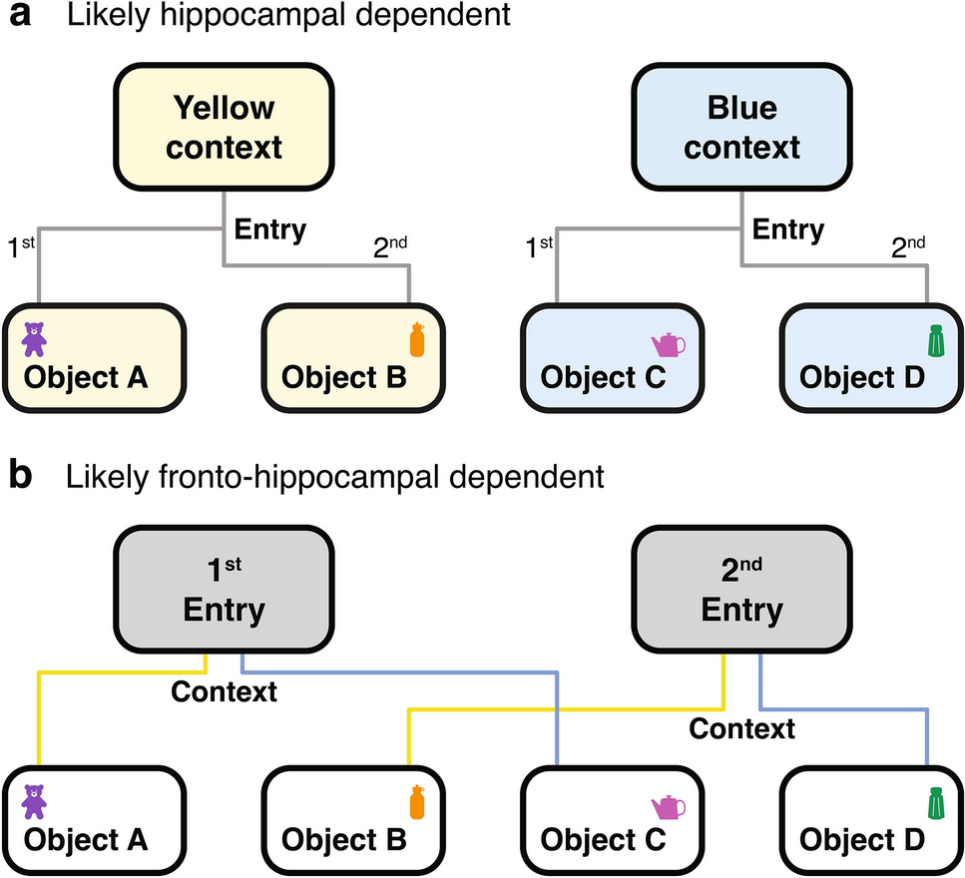
Schematic and heuristic model of potential memory pathways in single and adaptive context sequencing. (**a**) Two distinct memory pathways for Single context sequences, either in the yellow or blue context, where the physical context precedes the temporal order in which objects are remembered. This process may be hippocampal-dependent, with the context initiating the memory sequence. (**b**) Overlapping memory pathways for Two context sequences, where memories are categorised by the temporal order of context entry rather than the physical context identity (i.e., yellow or blue). The prefrontal cortex may facilitate adaptive multiple context sequencing by providing feedback to the hippocampus, allowing the correct object in the object-pair under the new context to be selected, rather than initiating the memory sequence anew based on the contextual change.

McKenzie et al.^31^ replicated the rodent object-context study conducted by Navawongse & Eichenbaum^25^ with the addition of electrophysiology of cells in CA1 and CA3 regions of the hippocampus. Their neurophysiological recording results suggest that context-dependent information is recalled in a hierarchical order, with the first being the context of the environment, followed by spatial position of the objects, the valence of each object, and finally, the item identity of the rewarded object. This schema organisation is suggested to explain how the rats were able to identify the correct object, even when the objects were identical across different contexts. We propose a key extension to these models and hypothesise that the neural substrates crucial for different aspects of this behaviour are likely found in the frontal-hippocampal circuit. The Single Context memory object-pairs may well rely on hippocampal-dependent processes (Fig. 6a); however, for adaptive multiple context memory pairing, as in Phase 2 (Two Context), it is likely that the prefrontal cortex accommodates changes in context via feedback to the hippocampal memory system. This would enable the selection of the correct object in the sequence under the new context, rather than the context change acting as a cue to initiate the memory sequence anew under the new context (Fig. 6b). The neuronal mechanisms and system interactions involved could now be studied, founded in the behavioural results here, at least in rats that can manage multiple changing contexts.

A key limitation of this study is the small sample size. Larger cohorts could be studied in the future to more accurately identify the proportion of rats capable of flexibly adapting their behaviour to multiple changing contexts. Nonetheless, this work provides a foundation for future studies, including the specific conditions that result in greater errors in the rodents as individuals or groups.

Another consideration is that the rats were initially trained on the Single Context object-pairs before being tested on the same phase. It remains unclear whether training them directly on the Two Context object-pairs from the outset would have facilitated better adaptation to context changes. It is possible that prior experience in the Single Context phase, rather than helping the rats, may have hindered many of them by reinforcing perseveration errors, making it more difficult for many of the rats to adjust when the Two Context condition was introduced. The errors observed in the rats that progressed to Phase 2 (Two Contexts) suggest that a shift in behavioural strategy was necessary. However, this adjustment was insufficient for four out of the six rats that reached that stage to achieve the performance required to progress further.

Moreover, the observed bias toward the blue context may indicate a preference for the darker environment, at least during initial training, potentially influencing performance. The relatively brighter yellow context may have been less familiar or less preferred, leading to reduced exploration and, consequently, impairing performance in the later phases for some of the rats. This potential environmental bias could have contributed to the difficulties most rats experienced in progressing through to the final stages of testing.

In an attempt to bridge the nonhuman and human animal literature, this study contributes to the development of a more comparable rodent model of human flexibility in context-guided behaviour. Our findings advance the understanding of the memory capabilities of rodents, particularly in adapting to changing contexts. It remains to be seen whether these results with rats could generalise and show individual variability with other species of rodents (e.g., mice), which also serve as important neurobiological animal models. These findings highlight the evolutionary foundations of context-guided memory and reveal remarkable individual variability in the ability of rats to flexibly navigate multiple contexts. Future experiments could investigate the neurobiological substrates and neuronal system mechanisms that underline the flexible behaviour demonstrated in the minority of the animals or explore the perseveration and other challenges in animals that struggle to accommodate changes in context – a trait also observed in the human population.

## Methods

### Subjects

Eight male Lister Hooded rats (*Rattus norvegicus*) supplied by Charles River, UK, were included in the study. All rats were housed in cages of three, in a room on a 12-h light–dark cycle from 07:00 to 19:00, with temperature (20 ± 1 °C) and humidity (55 ± 10%) kept stable. The cages in which the animals were housed (l: 56 × w: 38 × h: 22 cm, supplied by RC2F, NKP isotec., UK) included a red translucent tunnel that would later be used for transportation. Experimentation occurred during the light phase with food and water freely available throughout. Animals were not euthanised as part of the experiments. All procedures undertaken were in accordance with the guidelines of the UK Animals (Scientific Procedures) Act of 1986 and approved by Durham University AWERB and the Home Office (procedure licence number: PP8877096). The researchers working with the rats on this study had Home Office approved Personal Licenses to conduct the animal research reported. Reporting follows the recommendations of the ARRIVE guidelines.

### Apparatus

**Fig. 7:**
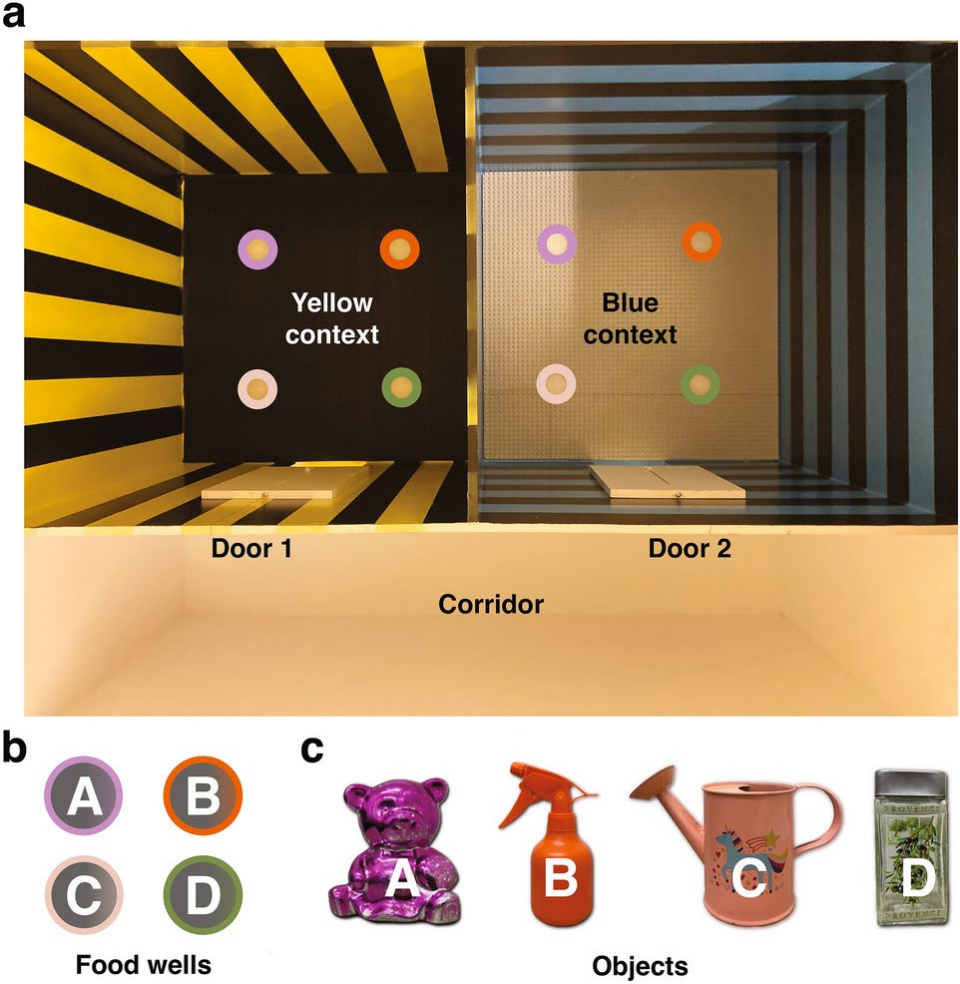
Aerial view of the maze, food wells and objects. (**a**) An aerial view of the maze, with two contexts—‘Yellow Context’ on the left and ‘Blue Context’ on the right. (**b**) Shows the orientation of the four wells within the maze. (**c**) Displays the arrangement of four objects placed consistently above the wells in each context: ‘A’ over the top-left well, ‘B’ over the top-right well, ‘C’ over the bottom-left well and ‘D’ over the bottom-right well.

The maze (Fig. 7a) consisted of two square contexts (l: 50 × w: 50 × h: 50 cm) joined to a corridor spanning the length of the two adjacent contexts (l: 100 × w: 25 × h: 50 cm). Each context was distinct with individually patterned walls and with removable inserts (l: 50 × w: 50 cm) that changed the texture of the floor. One insert was made of grey Lego, and the other from black rubber matting. The entire maze (l: 100 × w: 75 × h: 50 cm) was made of 10-mm-thick PVC foam sheet and painted using spray paint. Entrances to each context (l: 15 × w: 12 cm) were blocked by a movable door attached to the top of the maze by fishing string. The floors of each context included four circular wells (diameter: 4 cm × depth: 2 cm) spaced equidistantly from the four walls of the context and from one another (24 cm, Fig. 7a,b). The wells contained stainless steel cups (diameter: 4 cm × depth: 2 cm) that were removable. Each well was topped by one of four objects (of which there were three copies of each, Fig. 7c). All objects were distinct in colour, size, and shape. The maze itself was in a room with no obvious visual landmarks available to the animals inside the maze. The testing room was dimly lit (diffuse white light from a 100-W lamp) and had white noise playing in the background to prevent disturbance from external noises. Disinfectant wipes (Clinell universal wipes, GAMA Healthcare Ltd., UK) were used to clean all objects, floor inserts, and the maze. This consistently occurred after every trial. The thoroughness of cleaning was further validated through behavioural analyses comparing the performance on a given trial with that of the preceding trial (Supplementary Fig. 2). This comparison revealed no significant differences for the two main phases of the experiment (see Testing Procedure), suggesting that rats were not influenced by residual odours or markers from the previous trial.

### Habituation procedure

#### Handling

Rats were habituated to the handler and testing room prior to experimentation. Habituation began with three days of handling in the housing room, with each cage being handled for 15 min in total per day. During this time, each animal within the cage was picked up via the red translucent tube; this was intended to reduce unnecessary contact and promote a less stressful, less anxious environment^32^. The process was repeated for the duration of the session, with all animals being handled an equal number of times. Once acclimated to both the handler and the handling procedure, each cage of three rats was handled in the testing room for 10 min a day for six consecutive days.

#### Maze habituation

To introduce the rats to the maze, one of the three housing cages was brought to the testing room, and all rats inside were allowed to explore the entire maze as a group over the course of 30 min. Excretion levels were monitored to help identify if any of the rats were particularly anxious about being in the maze, and food pellets (45 mg LabTab MLab, Indiana, USA)the reward used later in the experimentwere generously scattered to promote exploration of all areas. After one day of group habituation, the animals were introduced to the maze individually and allowed to explore the entire maze uninterrupted. The rats were picked up using the red tube as indicated previously, and placed in the corridor of the maze with their heads facing the two doors. Similarly, the red tube was also implemented when removing the rats from the maze, during the tenth minute of the session, again with movement only occurring in the corridor. The procedure of moving the animals via the red tube was kept consistent throughout the experiment. Individual maze habituation was carried out over two days and was followed by two days of handling in the testing room to further solidify the importance of movement both in and out of the red transport tube.

#### Shuttling

Rats were trained to shuttle between the two contexts of the maze, with each animal participating in a 10-min session that was repeated over four days, within which they were guided to enter and leave a context by the use of food pellets. The doors of the maze were controlled by a string attached to a pulley that the handler could lift and lower at their own discretion. During a typical shuttling session, a pellet was placed in the centre of both contexts and a door to a context was opened, allowing the animal to enter and retrieve the pellet. Upon entering the context, the door was lowered and the animal remained inside. A pellet was then placed in the centre of the corridor before the door to the context was opened once more, and the animal was allowed to leave and retrieve the newly placed pellet. This was repeated several times during the session, with the context the animal was required to shuttle to and from being pseudo-randomly selected. Each animal was permitted to progress to the next level of habituation once 95% of the shuttle runs they performed within a 10-min session were under one minute in duration.

#### Object habituation

After successfully shuttling between contexts, the four objects the animals would encounter during the experiment were introduced individually. This involved each object being presented to the rat in the location that they would be kept in during testing. As seen in Fig. 7b,c, object A (“purple bear”) was placed in the top-left corner and object B (“orange pump”) was placed in the top-right corner. In the blue context, object C (“pink watering-can”) was placed in the bottom-left corner and object D (“green shaker”) was placed in the bottom-right corner. At first, a pellet was placed directly adjacent to the object to promote maximum interaction,but then, once the animal was familiar with the object, they were taught to retrieve the pellet from the well underneath the object. This was achieved by placing the correct object next to the well with a pellet inside and following each subsequent interaction, repositioning the object so that it incrementally covered the well. With the first few interactions, the animal could still see the pellet but had to push the object away in order to retrieve the pellet. By the time the object completely covered the well, the animal knew where the pellet was and how to obtain it. Over the course of four days, each object was presented individually beginning with object A on the first day and ending with object D on the fourth day. Each animal was required to shuttle in and out of the context as previously described, with progression to the training stage only occurring once 95% of the runs were under one minute long. Again, each habituation session lasted 10 min.

### Training procedure

Once the animals had learned to associate a food pellet with each of the four objects, training began.

#### One object in one context

To start, the rats were exposed to the two object-pairs associated with each context (i.e., objects A and B in the yellow context and objects C and D in the blue context). The aim was to teach the animal, that upon first entry, either object A or C was to be chosen, and then upon second entry, the correct object was either B or D. To ensure the animals learned the correct order, the only object present in a given context at any one time was the correct object. As such, each rat was taught to shuttle in and out of the same context, first collecting a pellet from under the object present on the left (object A if in the yellow context or C in the blue context), and then upon returning to the same context, from underneath the object on the right (object B in the yellow context and object D in the blue context). Each rat was allowed to explore the other wells and the context itself; however, if during a run the animal took longer than five minutes to retrieve a pellet, the run was aborted. All runs were counterbalanced, and each rat undertook six runs per day. This stage of training lasted a week, with each rat completing 36 runs in total.

#### One object in two contexts

Next, the rats were exposed to the two object-pairs associated across contexts (i.e., objects A and D going from yellow to blue context and objects C and B going from blue to yellow context). Much like the first training step, this was to teach the rats that object order could be split across contexts, and that entry into a context denoted the order. Again, only the correct object was present during an entry, and each rat had to shuttle between the two separate contexts, first collecting a pellet from the object present on the left in the first context, and then upon entering the second context, from underneath the object on the right. Similarly, all wells and the context were freely available to be explored; however, if the animal took longer than five minutes to obtain the pellet, the run was aborted.

#### Two objects in two contexts

By this point, all rats had learned that the object on the left yielded a reward upon first entry of a context, and then upon re-entry it was the object on the right that now yielded a reward. To ensure the animals were not simply reliant on only one object being present from which to collect their reward, both objects were presented at once, requiring the rats to decide which one was correct. To aid their learning, the correct object was baited, and the incorrect object was not. If the rats were incorrect in their choosing, they were removed from the maze by the handler before receiving the pellet and placed in the time-out cage for two minutes; the run being subsequently aborted.

### Testing procedure

#### Single context (Phase 1): temporal order in one context

For the first stage of testing, the rats undertook a version of the ‘Two objects in one context’ training step, this time with both wells baited to prevent the rats from using the odour of the pellet reward as a cue to the location of the correct response. Each rat performed six trials per day, and trials were counterbalanced to ensure equal testing in both the yellow and blue contexts. An example trial involved a door to one of the contexts being opened by the experimenter, and the animal having to knock over the object they deemed to be correct. If the context was yellow, this was object A (Fig. 1a). The animal then shuttled out of the context to the corridor, and the experimenter rebaited both objects before the animal was allowed to re-enter and select the second object (again if in the yellow context, the correct choice upon re-entry was object B). Alternatively, if a trial involved the blue context, the correct order of objects was object C followed by object D upon re-entry (Fig. 1b). If the rats were incorrect in their choosing, the handler removed them from the maze before they could retrieve any food, and placed in the time-out cage for two minutes aborting the trial. To move on to the next stage of testing, the total number of correct trials had to exceed 10 out of 12 trials over the course of two days (*p* =  0.019, binomial test, one-tailed), classified henceforth as the predefined criterion.

#### Two context (Phase 2): temporal order across two different contexts

Once successfully above threshold, the rats progressed to the second testing stage in which they had to remember the object-pairs across contexts. This was similar to the second training step where the rats were taught to shuttle between contexts, except now, two baited objects were present in each context to remove any bias caused by odour from the reward. As such, each rat needed to decide which of the two objects available was the correct choice depending on which context was entered. For example, a trial that transitioned from yellow to blue context (Fig. 1c) involved the animal shuttling from the yellow context—retrieving the pellet from beneath object A, out of a possible choice of object A or B—to the blue context where it retrieved the pellet from beneath object D (after visibly seeing objects C and D together). Conversely, if the trial began in the blue context, the rat needed to shuttle from the blue context to the yellow context, selecting object C in the blue context followed by object B in the yellow context (Fig. 1d). Initially, all wells were baited; however, once rats reached threshold (i.e., more than 10 correct out of 12 trials over two days), they were moved on to a version in which only the correct object(s) were baited. This was to assess whether any animals had a preference for context order. If the animals were again successful and reached threshold (10 out of 12 trials correct across two days), they then progressed to the final testing stage.

#### Two context—four objects (Phase 3): combined object and context order

The last testing stage utilised the rats’ movement between the two contexts and required them to select the correct object from among the four. All four objects were the same across the two contexts, both in identity and location. Only the correct object was baited, and the rats had to perform at or above threshold in order to progress to the final stage. The final testing stage was identical to that of the penultimate stage, except that the animal now had control over the order of context entry within a trial. This meant the rat could utilise any of the four logical object-pairs (Fig. 1e–h) they had learned throughout the experiment, provided that a correct decision was made on each entry. If not, they were removed from the maze without reward and the trial was aborted.

### Behavioural analyses

All statistical analyses and figure generation (Figs. 2, 4, and 5) were conducted using RStudio (R version 4.3.3, R Core Team 2023). Specifically, the ‘ggdist’ package was utilised to create the distribution plot shown in Fig. 2b^33^.

Error ratios, defined as the total number of a specific error type divided by the total number of trials performed by each rat (Fig. 3a), were calculated to compare performance across individuals during both the single context and two context phases of the experiment. To evaluate the impact of context and error type on error ratios, two linear mixed-effects models were fitted. Random intercepts for each rat were included to account for individual variability in baseline error rates. Fixed effects were tested using a type-III hypothesis test, ensuring that the contribution of predictors was assessed after accounting for shared variance with other factors. Results were considered significant at *p* < 0.05.

Prior to conducting comparisons, the normality of the data was assessed using the Shapiro–Wilk test applied to error ratios, and outliers were identified based on the interquartile range (*k* = 1.5). No deviations from normality were detected, and no outliers required removal. Post-hoc comparisons between groups were performed where relevant, and Cohen’s *d* was calculated to quantify effect sizes for differences between means.

## Supplementary Information

Supplementary Information.

## Data Availability

Data will be made available on the corresponding author’s Open Science Framework account.
