# Supplementary material for: Temporal context-guided memory capabilities in rodents
Source: Sci Rep. 2025 May 28;15:18753. doi: 10.1038/s41598-025-95410-2 (PMC12119930; doi:10.1038/s41598-025-95410-2)
Supplement: Supplementary file 1 — Supplementary Information. [file 41598_2025_95410_MOESM1_ESM.pdf]

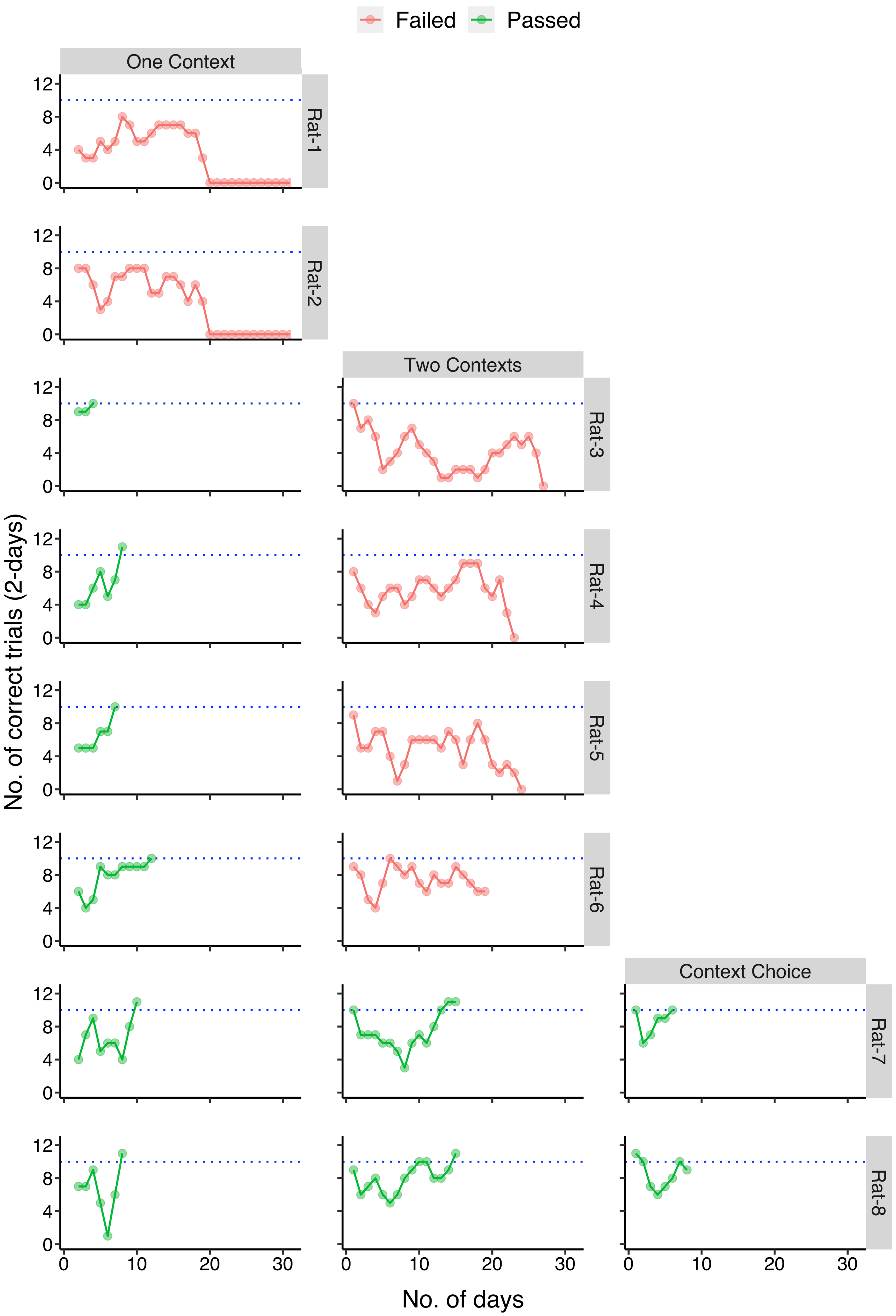
Failed
Passed
One Context
Two Contexts
Context Choice
Rat-1
Rat-2
Rat-3
Rat-4
Rat-5
Rat-6
Rat-7
Rat-8
No. of correct trials (2-days)
No. of days
0
4
8
12
0
10
20
30

Supplementary Figure 1. Number of trials needed to pass each testing stage for each rat.

The individual performance for each rat on the three testing stages, with the group split by those that passed (green - reached threshold – denoted by the dotted line - achieving 10/12 correct trials across two days) and failed (red).

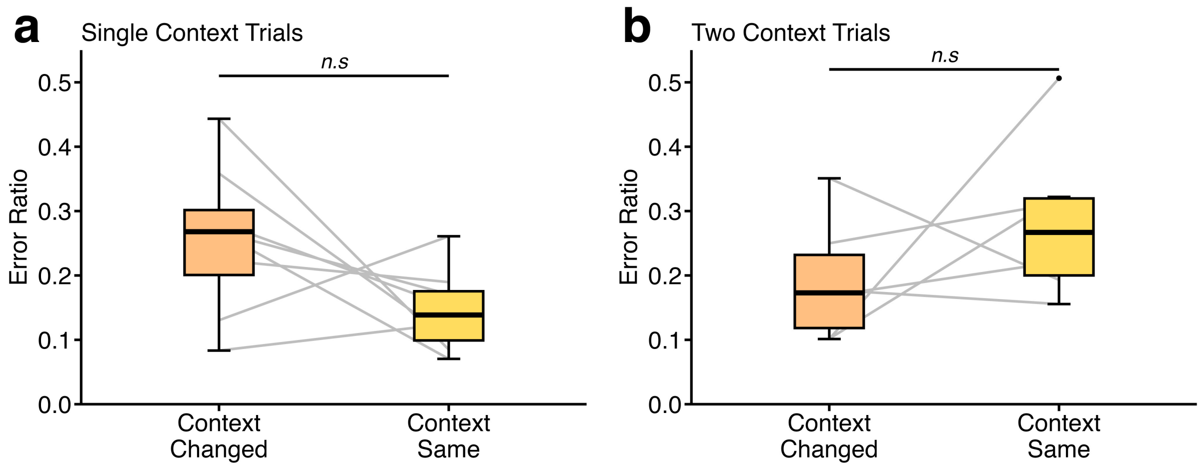

Supplementary Figure 2. Impact of the prior trial on error ratios across single and two context trials.

Error ratios based on whether the preceding trial changed ('Context Changed') or remained the same ('Context Same'), when trials were ordered chronologically in a session independent of rat. ***a)*** *In the Single Context phase (n = 8), no significant difference in error ratios was observed between trials when context changed compared to when the context remained the same (only a statistical trend: t (7) = 2.00, p = 0.085, d = 1.25, paired t-test).* ***b)*** *In the Two Context phase (n = 6), no significant difference in error ratios was found between trials where context changed versus when it remained the same (t (5) = 1.17, p = 0.296, d = 0.84, paired t-test). Individual animal trajectories are represented by lines connecting paired data points, illustrating within-subject performance.*
